# Association of ALDH3A1 expression with tumor differentiation, pathological stage, and nodal status in oral squamous cell carcinoma

**DOI:** 10.1016/j.jtumed.2025.06.007

**Published:** 2025-06-24

**Authors:** Rida Fatima Khan, Shazia Akbar Ansari, Uzma Bukhari, Muhammad Taqi, Sadia Farooqi, Ammar Ali Muhammad

**Affiliations:** a Department of Oral Pathology, Dow Dental College, Dow University of Health Sciences, Pakistan; b Department of Oral Pathology, Dow Dental College, Dow University of Health Sciences, Karachi, Pakistan; c Department of Pathology, Dow International Medical College, Dow Diagnostic Research and Reference Laboratory (DDRRL), Dow University of Health Sciences, Karachi, Pakistan; d Department of Community Dentistry, Dow Dental College, Dow University of Health Sciences, Karachi, Pakistan; e Department of Dentistry, Sindh Government Hospital, Karachi, Pakistan; f Department of Global Surgery, Interactive Research Department, Karachi, Pakistan

**Keywords:** سرطان الخلايا الحرشفية الفموي, بروتين ألدهيد ديهيدروجينيز ٣أ۱, الكيمياء المناعية، العلامة الحيوية, علامة الورم., ALDH3A1 protein, Biomarker, Immunohistochemistry, Oral squamous cell carcinoma (OSCC), Tumor marker

## Abstract

****Objective**:**

To observe the association between Aldehyde dehydrogenase 3 family member A1 (ALDH3A1) levels with histological grade, pathological stage and nodal status in Oral Squamous Cell Carcinoma (OSCC).

**Methods:**

A total of 114 diagnosed OSCC patients who underwent Excisional biopsy with complete neck dissection were recruited in the study in accordance with inclusion criteria. H & E slides were assessed for evaluation of clinicopathological features. Immunohistochemical (IHC) staining method was used to study the expression of ALDH3A1. Statistical analysis was performed using SPSS version 21.0. The chi-square test and Fisher exact test were applied to evaluate the association of ALDH3A1 expression with histological grade, nodal metastasis and pathological tumor stage (pT). All tests were two-sided and a *p*-value of <0.05 was considered significant.

**Results:**

This study looked at 114 OSCC patients, most of them were men (76.3 %) with a mean age of 47. The common tumor sites were buccal mucosa (53.5 %) and left side predominance of face (52.6 %). The grades were well (30.7 %), moderate (38.6 %), and 3 poorly differentiated (30.7 %). The expression of ALDH3A1 is significantly strong in the well differentiated compared to the poorly differentiated OSCC (*p* < 0.001), and presence of nodes involvement compared to no node’s involvement (*p* < 0.001). The findings also suggest that ALDH3A1 expression decreases in more advanced tumors (pT4 stage, *p* = 0.012), with higher ALDH3A1 levels being associated with earlier stages (pT1, pT2) of OSCC.

**Conclusions:**

The decrease in expression of ALDH3A1 in a poorly differentiated and advanced stage OSCC compared to its well differentiated and early stage highlights the potential role of this biomarker as an important prognostic tool.

## الملخص

**أهداف البحث:**

إنزيم ألدهيد ديهيدروجينيز ٣ من عائلة أ۱ هو إنزيم ألدهيد ديهيدروجينيز، وهو علامة على الخلايا الجذعية السرطانية ومؤشر على تشخيص أنواع مختلفة من السرطان. في هذه الدراسة، نرصد العلاقة بين مستويات إنزيم ألدهيد ديهيدروجينيز ٣ من عائلة أ۱ والدرجة النسيجية والمرحلة المرضية وحالة العقد اللمفاوية في سرطان الخلايا الحرشفية الفموي باستخدام الكيمياء المناعية.

**طريقة البحث:**

تم اختيار ۱۱٤ مريضا مصابا بسرطان الخلايا الحرشفية الفموي، والذين خضعوا لخزعة استئصالية مع تشريح كامل للعنق، للمشاركة في الدراسة وفقا لمعايير الإدراج. قيمت شرائح الهيماتوكريت والإستروجين لتقييم السمات السريرية والمرضية. واستخدمت طريقة التلوين الكيميائي المناعي لدراسة التعبير عن إنزيم ألدهيد ديهيدروجينيز ٣ من عائلة أ۱. تم تطبيق اختبار مربع كاي واختبار فيشر الدقيق لتقييم ارتباط التعبير عن جين أ۱ من عائلة ألدهيد ديهيدروجينيز ٣ مع الدرجة النسيجية، ونقائل العقد اللمفاوية، ومرحلة الورم المرضية (بي تي). كانت جميع الاختبارات ثنائية الجانب، واعتُبرت القيمة الاحتمالية > ۰٬۰٥ ذات دلالة إحصائية.

**النتائج:**

تناولت هذه الدراسة ۱۱٤ مريضا بسرطان الخلايا الحرشفية الفموي، معظمهم من الرجال (٧٦٬٣٪) بمتوسط عمر ٤٧ عاما. كانت مواقع الورم الشائعة هي الغشاء المخاطي للفم (٥٣٬٥٪)، وغلبة الجانب الأيسر من الوجه (٥۲٬٦٪). كانت الدرجات جيدة (٣۰٬٧٪)، ومتوسطة (٣۸٬٦٪)، وضعيفة التمايز (٣۰٬٧٪). كان التعبير عن إنزيم ألدهيد ديهيدروجينيز ٣ من عائلة أ۱ قويا بشكل ملحوظ في سرطان الخلايا الحرشفية الفموي جيد التمايز مقارنة بسرطان الخلايا الحرشفية الفموي ضعيف التمايز، ووجود إصابة في العقد اللمفاوية مقارنة بعدم إصابة العقد اللمفاوية. تشير النتائج أيضا إلى أن التعبير عن إنزيم ألدهيد ديهيدروجينيز ٣ من عائلة أ۱ ينخفض في الأورام الأكثر تقدما (مرحلة بي تي ٤)، مع ارتباط ارتفاع مستويات إنزيم ألدهيد ديهيدروجينيز ٣ من عائلة أ۱ بالمراحل المبكرة (بي تي ۱، بي تي ۲) من سرطان الخلايا الحرشفية الفموي.

**الاستنتاجات:**

يبرز انخفاض التعبير عن إنزيم ألدهيد ديهيدروجينيز ٣ من عائلة أ۱ في سرطان الخلايا الحرشفية الفموي في مرحلة متقدمة وقليلة التمايز، مقارنة بمرحلته المبكرة جيدة التمايز، الدور المحتمل لهذا المؤشر الحيوي كأداة تشخيصية مهمة.

## Introduction

According to Global Cancer Statistics (GLOBOCAN 2022), lip and oral cavity cancer ranks as the 16th most common malignancy worldwide, with 389,846 new cases reported in 2022, and it is the 15th highest cause of cancer-related deaths, accounting for 188,438 fatalities. The five-year prevalence of lip and oral cavity cancer is 1,094,448 cases globally.^1^ In particular, the incidence of oral squamous cell carcinoma (OSCC) is rising and is projected to increase by 30 % beyond 2030.^2^ South and Southeast Asia have the highest rates of OSCC occurrence, with significant regional variations.^3^ Data from the Dow Cancer Registry in Karachi indicate that OSCC is the most frequently diagnosed cancer among males and the second most common cancer among females in the region.^4^ The widespread use of substances such as cigarettes, alcohol, and betel quid contribute to these high rates, and all are recognized as major risk factors for OSCC.^5^^,^^6^

Despite advances in the detection, prevention, and treatment of OSCC, the prognosis remains concerning, where the five-year survival rate is significantly impacted by the stage at diagnosis. Survival rates range from approximately 90 % for early stage OSCC to around 30 % for advanced stages.^7^, ^8^, ^9^ This difference highlights the critical importance of early diagnosis.

Tissue biopsy remains the gold standard for diagnosing OSCC because it provides reliable information about the tumor's clinicopathological characteristics, histological grade, and extent of spread.^10^ However, the conventional biopsy methods have limitations in terms of predicting tumor behavior and patient prognosis. To address these shortcomings, biomarkers have emerged as pivotal tools in cancer diagnostics, offering insights into tumor progression, aggressiveness, and resistance to treatment.^11^^,^^12^

Reactive oxygen species (ROS) are highly reactive molecules containing unpaired electrons, and they play dual roles in cellular dynamics.^13^ ROS support growth and differentiation at low levels, whereas they promote tumor survival and angiogenesis while at high concentrations.^14^ This dual role highlights ROS as potential therapeutic targets in cancer treatment.^15^, ^16^, ^17^

Aldehyde dehydrogenase (ALDH) enzymes have important roles in the management of ROS and are also considered cancer stem cell markers due to their high expression levels in cancer. ALDH enzymes are responsible for the detoxification of aldehydes and reduction of oxidative stress, thereby promoting cellular protection. The elevated activities of ALDH enzymes in cancer stem cells are linked to tumor progression and poor prognosis, and thus they are potential therapeutic targets.^18^^,^^19^

In the ALDH family, ALDH3A1 plays a key role in oxidizing a broad range of endogenous and exogenous aldehydes, and it is essential for critical cellular processes such as proliferation, differentiation, and survival in both normal and cancerous cells.^20^^,^^21^

Overexpression of ALDH3A1 has been observed in various cancers, including those affecting the oral cavity, esophagus, nasal structures, and minor salivary glands.^22^, ^23^, ^24^ ALDH3A1 was recently identified as a marker of cancer stem cells that have positive correlations with chemoresistance, tumor progression, and poor clinical outcomes in the context of cancer. ALDH3A1 downregulation in OSCC was demonstrated in previous studies with molecular techniques, and its reduced expression was linked to epithelial mesenchymal transition and inflammation. However, although lower ALDH3A1 levels were found in tumor tissue, previous studies did not determine the detailed immunohistochemical status of ALDH3A1 expression according to the histological grade, tumor stage, or lymph node metastasis.^23^ Thus, in the present study, we aimed to address the need to conduct a comparative analysis of ALDH3A1 expression and the key clinical pathological parameters, i.e., histological grade, tumor stage, and lymph node metastasis. We analyzed ALDH3A1 protein expression by immunohistochemical staining and assessed the relationships with the histological grade, tumor stage, and lymph node metastasis in OSCC patients, thereby allowing us to obtain greater clinical insights into ALDH3A1 than previous biological findings, and to leverage its prognostic potential for OSCC. Thus, in this study, we determined the associations between ALDH3A1 expression and clinicopathological features in OSCC to assess its potential role as a prognostic biomarker.

## Materials and Methods

This cross-sectional analytical study was based on 114 formalin-fixed, paraffin-embedded (FFPE) OSCC tissue blocks. All samples that satisfied the inclusion criteria were selected. The sample size was calculated using OpenEpi version 3.01 based on the ALDH3A1 expression rates reported by Qu et al. (55.6 % positive expression in OSCC samples).^23^ The study was conducted in the Histopathology Section of Dow Diagnostic Research and Reference Laboratory, Dow University of Health Sciences (DUHS), Karachi, Pakistan from September 2021 to May 2023. Ethical approval was obtained from the Institutional Review Board (IRB) of DUHS (IRB Number: IRB-2566/DUHS/Approval/2022/1007).

The inclusion criteria comprised excisional biopsy specimens of OSCC tissue and neck dissection samples with at least 70 % full thickness section, regardless of the patient's age or gender. Exclusion criteria included samples with inconclusive diagnoses, inadequate fixation, recurrent tumors, prior chemotherapy or radiotherapy, and individuals with chronic illnesses, autoimmune diseases, or congenital syndromes.

### Tissue processing and clinicopathological assessment

Demographic data and clinicopathological details were collected after obtaining informed verbal consent from all patients. OSCC tissue specimens were fixed in 10 % neutral-buffered formalin. Two adjacent non-tumorous oral epithelium tissue specimens were used as controls.^23^ The diagnosis of OSCC was confirmed by histopathological examination of slides stained with hematoxylin and eosin. Histological grading and pathological tumor (pT) node metastasis staging were assessed by two independent histopathologists (UB and RF). Histological grading was performed using Broder's classification by categorizing the samples as Grade 1 (G1): well-differentiated, Grade 2 (G2): moderately differentiated, and Grade 3 (G3): poorly differentiated.^25^

Pathological staging was conducted according to the pT node metastasis classification outlined in the 8th edition (2017) of the AJCC/UICC TNM staging guidelines. This system incorporates tumor size and depth of invasion for T staging, and lymph node involvement and extracapsular spread for N staging.^25^

### Immunohistochemistry

To conduct the immunohistochemical analyses, full thickness OSCC tissue sections were cut into slices with a thickness of 4 μm and mounted on positively charged slides to ensure good adhesion. The slides were dried in an oven at 70 °C for 1 h. Xylene was used for deparaffinization with three immersions each for 5 min, before rehydrating with a graded series consisting of 70 %, 80 %, and 100 % methanol, and then washing with distilled water until the residual methanol was removed. Endogenous peroxidase activity was blocked by using 3 % hydrogen peroxide for 5 min in order to minimize background staining.

Antigen retrieval was performed by utilizing Tris buffer (pH 9.0) in a pressure cooker for 20 min, before cooling at room temperature for 30 min. Slides were washed three times with phosphate-buffered saline (PBS), before applying the primary antibody for ALDH3A1 (Anti-Rabbit polyclonal, Thermo Fisher CAT #PA5-15004) at a dilution of 1:500 in PBS with 1 % bovine serum albumin (BSA) solution. The sections were incubated with the primary antibody for 60 min at room temperature and then washed three times with PBS to remove any unbound antibody.

Horseradish peroxidase-labeled secondary antibody (Thermo Fisher CAT #PI31490) was then applied at a dilution of 1:1000 in PBS with 1 % BSA, and incubated for 30 min. After washing three times with PBS, 3,3′-diaminobenzidine was applied as the chromogen substrate and the brown color indicated formation of the antigen–antibody complex. The slides were counterstained with hematoxylin to observe the nucleus. Slides were then dried in an oven and mounted with dibutylphthalate polystyrene xylene to allow their permanent preservation. ALDH3A1 expression was analyzed based on these stained sections.

### Immunoreactivity scoring (IRS) method

The semi-quantitative IRS method was used to analyze ALDH3A1 protein expression in the OSCC tissue biopsy specimens. The percentage of positive cells was determined by manual counting and reevaluated using Fiji ImageJ software. The software was used to apply a digital analysis method that supports IRS scoring and helps to reduce observer bias and increase intra- and inter-examiner reliability.^26^ Immunoreactivity was scored on a scale from 0 to 4 based on the number of positive cells, with a score of 0 for no positive cells, 1 for less than 10 % positive cells, 2 for 10–50 % positive cells, 3 for 50–80 % positive cells, and 4 for more than 80 % positive cells (Table 1). The intensity of staining was evaluated using a microscope by two independent histopathologists (UB and RF). The scores were categorized based on the intensity of the staining as follows: a score of 0 for no staining, 1 for weak staining, 2 for moderate staining, and 3 for strong staining. The final IRS score was calculated by multiplying the score for the percentage of positive cells by the staining intensity score. The final scores were categorized as follows: scores of 0–1 indicated negative expression, scores of 2–3 denoted weak expression, scores of 4–8 represented moderate expression, and scores of 9–12 indicated strong expression.^27^

**Table 1:** Immunoreactivity scoring method for assessing immunohistochemical staining.

	Staining intensity	(percentage of positive cells × staining intensity) = immunoreactivity score
**Score 0: no positive cells**	Score 0: No staining	0–1 = negative
**Score 1: < 10 % positive cells**		
**Score 2: 10–50 % positive cells**	Score 1: Weak	2–3 = weak expression
**Score 3: 50–80 % positive cells**	Score 2: Moderate	4–8 = moderate expression
**Score 4: > 80 % positive cells**	Score 3: Strong	9–12 = strong expression

### Statistical analysis

Data analysis was performed using SPSS software version 21.0. Continuous variables, such as age, were summarized as the mean ± standard deviation, whereas categorical variables, including gender, histopathological tumor grades (G1, G2, and G3), presence of lymph node metastases, lesion site, tumor laterality, and pathological stage of OSCC, were represented as frequencies and proportions.

The chi-squared test was employed to evaluate the associations between ALDH3A1 expression and histological grades of OSCC, nodal metastases, pT staging, and other categorical variables. Fisher's exact test was used as when the criteria for the chi-squared test were not satisfied. All statistical tests were two-sided, and a *p*-value <0.05 was considered to indicate a statistically significant difference.

## Results

### Clinicopathological characteristics of OSCC

This study included 114 OSCC tissue specimens. The most common tumor site was the buccal mucosa in 61 cases (53.5 %), followed by the tongue in 20 cases (17.5 %), lip in 14 cases (12.3 %), and alveolus of the mandible in 10 cases (8.8 %). Less frequent sites included the floor of the mouth, alveolus of the maxilla, retromolar area, and palate, with two cases for each (1.8 %), and the gingiva in one case (0.9 %) (Table 2).

**Table 2:** Clinicopathological characteristics of OSCC cases.

	Parameter	Frequency N (%)
**Tumor site**	Buccal mucosa	61(53.50)
	Floor of the mouth	2 (1.80)
	Lip	14 (12.30)
	Tongue	20 (17.50)
	Alveolus of mandible	10 (8.80)
	Alveolus of maxilla	2 (1.80)
	Retromolar area	2 (1.80)
	Palate	2 (1.80)
	Gingiva	1 (0.90)
**Tumor laterality**	Right	32 (28.10)
	Left	60 (52.60)
	Midline	8 (7.00)
	Not specified	14 (12.30)
**Histological grade**	Well differentiated	35 (30.70)
	Moderately differentiated	44 (38.60)
	Poorly differentiated	35 (30.70)
**Pathological tumor stage**	pT1	31 (27.20)
	pT2	30 (26.30)
	pT3	26 (22.80)
	pT4	27 (23.70)
**Nodal metastasis**	No lymph node metastasis	69 (60.50)
	Lymph node metastasis	45 (39.50)

In terms of tumor laterality, most tumors were located on the left side, accounting for 60 cases (52.6 %), with 32 cases (28.1 %) on the right side. Tumors involving the midline were identified in eight cases (7.0 %), and unspecified laterality in 14 cases (12.3 %) (Table 2).

Histological grading showed that 35 cases (30.7 %) were well differentiated, 44 cases (38.6 %) were moderately differentiated, and 35 cases (30.7 %) were poorly differentiated. According to pT staging analysis, 31 cases (27.2 %) were classified as pT1, 30 cases (26.3 %) as pT2, 26 cases (22.8 %) as pT3, and 27 cases (23.7 %) as pT4. Nodal metastasis was absent in 69 cases (60.5 %), but lymph node metastases were found in 45 cases (39.5 %). Overall, comprehensive distributions of tumor sites, laterality, histological grading, pathological staging, and nodal involvement were analyzed for the OSCC cases (Table 2).

### Correlations of ALDH3A1 expression with histological grade, pathological stage, and lymph node metastasis

The objective of our study was to determine the associations of ALDH3A1 with the histological grade, pathological stage, and nodal status of OSCC samples. The IRS results for ALDH3A1 indicated a significant association with the advanced histological grade and stage, and the presence of nodal metastasis in patients with OSCC. The results are summarized in Table 3.

**Table 3:** Distribution of ALDH3A1 immunoreactivity scoring (IRS) results according to clinicopathological variables in OSCC patients.

IRS results for ALDH3A1 expression					
Variables	Weak N (%)	Moderate N (%)	Strong N (%)	Total N (%)	*p*-value
**Gender**					
Male	30 (26.3)	50 (43.9)	7 (6.1)	87 (76.3)	0.352
Female	11 (9.6)	16 (14.1)	0 (0)	27 (23.7)	
**Age (years)**					
22–25	6 (5.3)	1 (0.9)	0 (0)	7 (6.1)	0.331
26–45	16 (14)	28 (24.6)	4 (3.5)	48 (42.1)	
46–75	18 (15.8)	34 (29.9)	3 (2.6)	55 (48.3)	
76–84	1 (0.9)	3 (2.6)	0 (0)	4 (3.5)	
**Tumor site**					
Buccal mucosa	19 (16.6)	37 (32.4)	5 (4.3)	61 (53.3)	0.615
Floor of the mouth	0 (0)	2 (1.8)	0 (0)	2 (1.8)	
Lip	7 (6.1)	6 (5.3)	1 (0.9)	14 (12.3)	
Tongue	8 (7)	12 (10.5)	0 (0)	20 (17.5)	
Alveolus of mandible	4 (3.5)	6 (5.3)	0 (0)	10 (8.8)	
Alveolus of maxilla	1 (0.9)	0 (0)	1 (0.9)	2 (1.8)	
Retromolar area	1 (0.9)	1 (0.9)	0 (0)	2 (1.8)	
Gingiva	0 (0)	1 (0.9)	0 (0)	1 (0.9)	
Palate	1 (0.9)	1 (0.9)	0 (0)	2 (1.8)	
**Tumor laterality**					
Right	9 (7.9)	22 (19.3)	1 (0.9)	32 (28.1)	0.426
Left	23 (20.2)	33 (28.9)	4 (3.5)	60 (52.6)	
Midline	5 (4.4)	3 (2.6)	0 (0)	8 (7)	
Not specified	4 (3.5)	8 (7)	2 (1.8)	14 (12.3)	
**Pathological stage**					
pT1	4 (3.5)	22 (19.3)	5 (4.4)	31 (27.2)	0.012^a^
pT2	15 (13.2)	14 (12.3)	1 (0.9)	30 (26.3)	
pT3	11 (9.6)	14 (12.3)	1 (0.9)	26 (22.8)	
pT4	11 (9.6)	16 (14)	0 (0)	27 (23.7)	
**Histological grade**					
G1	0 (0.0)	31 (27.2)	4 (3.5)	35 (30.7)	<0.001^a^
G2	14 (12.3)	27 (23.7)	3 (2.6)	44 (38.6)	
G3	27 (23.7)	8 (7.0)	0 (0.0)	35 (30.7)	
**Regional lymph node metastases**					
No lymph node metastases	9 (7.9)	53 (46.5)	7 (6.1)	69 (60.5)	<0.001^a^
Tumor with lymph node involvement	32 (28.1)	13 (11.4)	0 (0.0)	45 (39.5)	

a Significant.

According to the histological grade, strong ALDH3A1 expression was observed in four well differentiated OSCC cases (11.4 %), whereas moderate ALDH3A1 expression was found in the majority cases (31/35; 88.5 %). Weak expression was not observed in any case in this group. In moderately differentiated OSCC cases, weak expression was observed in 14 cases (31.8 %), moderate expression in 27 cases (61.4 %), and strong expression in three cases (6.8 %). ALDH3A1 expression was weak in poorly differentiated tumors (27/35; 77.1 %) and moderate in eight cases (22.9 %), but strong expression was not observed.

**Figure 1:**
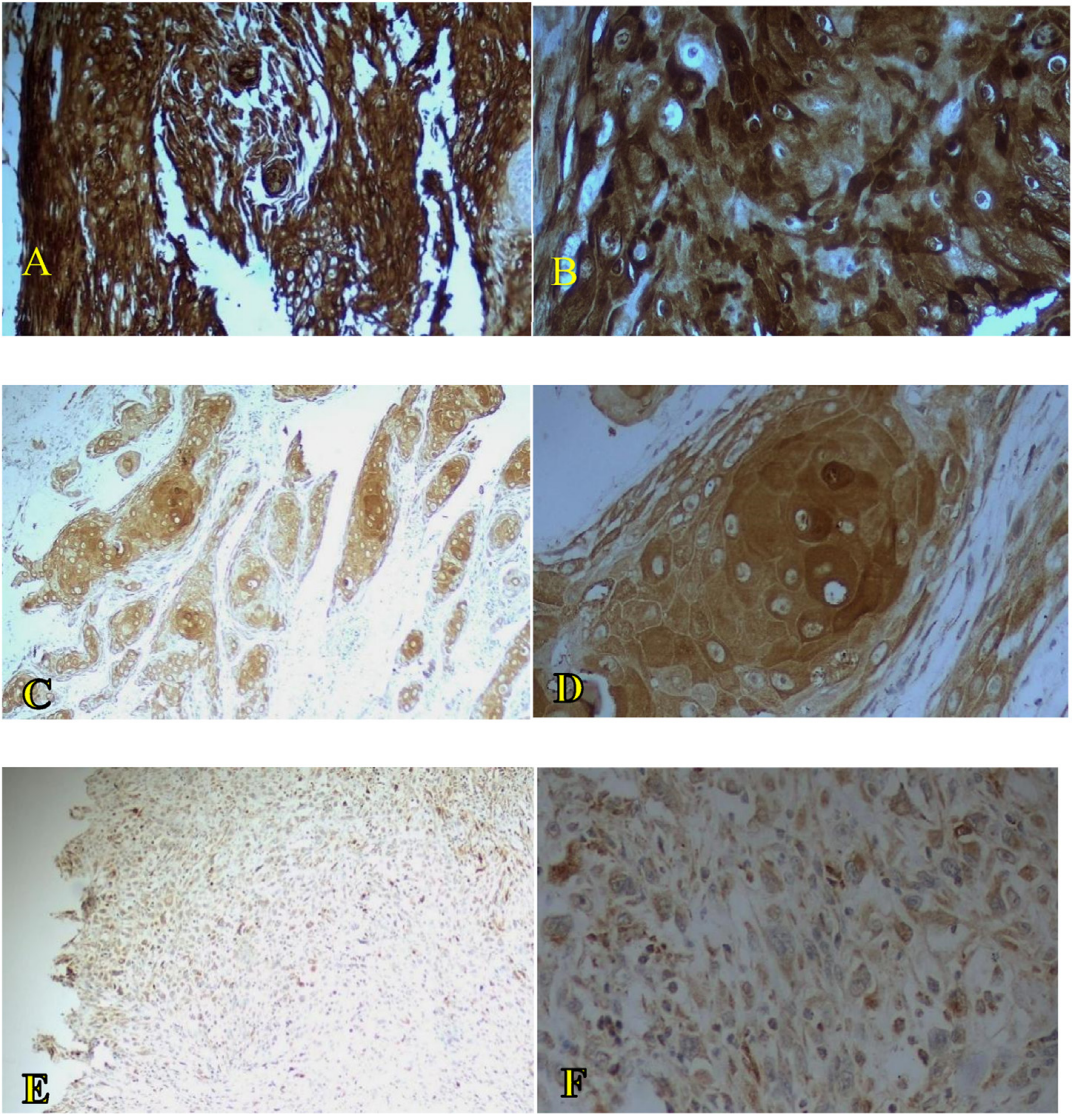
**Images of ALDH3A1 immunohistochemical staining based on immunoreactivity scoring method for histological grades G1, G2, and G3 at magnifications of 10 × and 40 × .A and B:** Well differentiated (G1) OSCC showing strong expression at magnifications of 10 × and 40 × , respectively. **C and D:** Moderately differentiate (G2) OSCC showing moderate expression at magnifications of 10 × and 40 × , respectively. **E and F:** Poorly differentiated (G3) OSCC showing weak to moderate expression at magnifications of 10 × and 40 × , respectively.

A statistically significant association was found between ALDH3A1 expression and histological grade (Pearson's chi-square = 46.278, df = 4, *p* < 0.001). These findings indicate that there was a strong inverse association between ALDH3A1 expression and tumor dedifferentiation, where decreased ALDH3A1 expression was correlated with increased histological grades. Therefore, these findings imply a decrease in ALDH3A1 expression as the tumor grade advanced (Figure 1).

In terms of the association with nodal metastasis, most tumors without lymph node involvement exhibited moderate ALDH3A1 expression (53/69; 76.8 %), whereas weak (nine; 13.0 %) and strong (seven, 10.2 %) expression levels were observed in other cases. By contrast, in cases with nodal metastasis involvement, weak expression was found in 32 of 45 cases (71.1 %) and moderate expression in 13 cases (28.9 %), and it is important to note that no cases with nodal metastasis expressed strong levels of ALDH3A1. These findings indicate a decrease in ALDH3A1 expression in tumors with nodal metastases and poorly differentiated OSCC.

Evaluation of ALDH3A1 expression across pT stages also indicated a statistically significant association (Pearson's chi-square = 15.643; df = 6; *p* = 0.012). In early-stage tumors (pT1), moderate expression was predominant (22/31; 70.9 %), followed by strong (five cases; 16.1 %) and weak expression (four cases; 13 %). In pT2 tumors, half of the cases (15/30; 50 %) exhibited weak expression, with moderate expression in 14 cases (46.7 %) and strong expression in only one case (3.3 %). In pT3 tumors, moderate expression was most common (14/26; 53.8 %), followed by weak (11 cases; 42.3 %) and strong (one case; 3.9 %) expression. In advanced pT4 tumors, moderate expression remained predominant (16/27; 59.3 %), but weak expression was found in 11 cases (40.7 %), and no strong staining was observed. Overall, these data indicate that ALDH3A1 expression declined as the tumor stage advanced, where weak expression became more frequent in later stages.

These results indicate that ALDH3A1 may serve as a potential marker for early-stage tumors and predictive indicator of lymph node involvement, contributing to the assessment of OSCC prognosis and progression.

However, no statistically significant differences in ALDH3A1 expression levels were observed according to variables such as age, gender, tumor site, and tumor laterality, as shown in Table 3.

## Discussion

ALDH3A1, a member of the ALDH enzyme family, is important for homeostasis of the oral epithelial by detoxification, retinoic acid biosynthesis, and regulating cellular differentiation.^28^ In normal oral mucosa, ALDH3A1 expression is mainly observed in the upper suprabasal layers, and it is responsible for terminal differentiation of keratinocytes and protection from environmental and oxidative stresses.^29^ Thus, the spatial expression pattern of ALDH3A1 indicates its importance for maintaining the structural and functional integrity of the oral epithelium. ALDH3A1 expression is mutated in an increasing number of cancers, particularly OSCC, but its loss or decrease may reflect altered differentiation and it could also be a marker of tumorigenesis.^30^, ^31^, ^32^, ^33^, ^34^ Recently, ALDH3A1 was identified as a promising prognostic biomarker in different malignancies, and it was correlated with diverse clinical outcomes in various cancers.^35^^,^^36^ However, the role of ALDH3A1 in OSCC is poorly understood and previous studies were inconclusive or found non-significant associations of ALDH3A1 with prognostic parameters.^23^

Thus, in the present study, we assessed the immunohistochemical expression levels of ALDH3A1 in OSCC tissue specimens and determined the correlations with major clinicopathological parameters to explore its potential as a prognosis marker. We found significant associations between the ALDH3A1 protein expression levels in OSCC and the histological grade, pT stage, and lymph node metastasis. Overall, our findings suggest that there is a close relationship between ALDH3A1 expression and tumor progression and biological aggressiveness.

Our results showed that the ALDH3A1 expression level tended to decrease as the tumor grade advanced in OSCC (*p* < 0.001; Table 3). The ALDH3A1 expression levels were predominantly moderate to strong in well differentiated tumors but weak in poorly differentiated tumors, and strong expression was found in no low scoring cases. In addition, we found that weak ALDH3A1 expression was correlated with lymph node metastasis (*p* < 0.001). Moderate ALDH3A1 expression was predominant in cases without nodal involvement, and strong ALDH3A1 expression was found in none of the cases with nodal metastasis (Table 3). In addition, we found a significant inverse relationship between ALDH3A1 expression and tumor stage (pT). Weak ALDH3A1 expression was more frequent in higher stage tumors (pT3 and pT4), but moderate to strong expression was frequent in early-stage tumors (pT1) (Table 3).

These results are consistent with the study by Qu et al. (2020) who found a significantly lower ALDH3A1 expression in OSCC tissues compared with adjacent normal tissues. They also showed that the ALDH3A1 levels were significantly lower in patients with lymph node metastasis and poorer overall survival, with a similar *p*-value (0.037∗) to that in our study (*p* < 0.001). However, the association of ALDH3A1 with histological differentiation and staging was not significant. By contrast, we found that ALDH3A1 expression had inverse correlations with histological grade and pathological stage, with statistically significant *p*-values of <0.001 and < 0.012, respectively.

These differences in the statistical significance of the relationships with histological grade and pathological stage may have been due to differences in the sample size, study population, or criteria used for scoring ALDH3A1 immunoreactivity. However, both studies support the possible prognostic role of ALDH3A1 in OSCC due to its potential for predicting the metastatic potential and disease progression.^23^

These findings validate the results reported by Luo et al. (2024) who showed that chronic restraint stress promoted OSCC progression via increased norepinephrine secretion and ADRB2 expression, as well as decreased ALDH3A1 levels. These effects were linked to downregulated energy metabolism and increased mitochondrial activity. By contrast, ALDH3A1 overexpression inhibited OSCC cell proliferation, the epithelial–mesenchymal transition process, and mitochondrial metabolism. Moreover, they showed that reduced ALDH3A1 expression enhanced the polarization and metastasis of tumor-associated neutrophils (TANs) in head and neck squamous cell carcinoma, and played a central role as a tumor suppressor and metabolic regulator.^37^

In addition, the results obtained by He et al. agree with our findings, where they demonstrated that ALDH3A1 expression was inversely associated with infiltration by TANs. Higher TAN infiltration was positively associated with lower tumor differentiation (*p* = 0.005) and lymph node metastasis (*p* = 0.029), and significantly higher levels of TANs were observed in OSCC tissues.^38^

In contrast to the tumor grade, stage, and metastasis, we did not find strong associations between ALDH3A1 expression and demographic variables, tumor site, laterality, or clinical staging, thereby suggesting that ALDH3A1 has a stronger biological relationship with the tumor rather than anatomical or demographic factors when predicting outcomes.

We also evaluated clinicopathological parameters including age, gender, tumor site, and laterality. Most cases occurred in males, with 76.3 %, and the mean patient age was 47 ± 2 years. Similarly, Ullah et al. (2023) reported that 73.3 % of OSCC cases were in males, with a mean age of 47 years.^39^ By contrast, Bhuyan et al. (2022) observed a higher prevalence of OSCC among females aged 45–64 years in Eastern India, although males still comprised a significant proportion of the cases.^40^ This regional variation highlights the potential effects of cultural practices and exposure to risk factors on the distribution of OSCC.

Our assessment of the anatomical distribution showed that the buccal mucosa (53.5 %) and tongue (17.5 %) were the most commonly reported sites for OSCC. Mohanta et al. (2013)^41^ and Jyoti et al. (2020)^42^ reported that 45.2 % and 42 % of OSCC cases occurred in the buccal mucosa of patients, respectively. Moreover, our findings showed that the tongue was the second most frequently affected site, as also reported by Gupta et al. (2021) who identified both the tongue and buccal mucosa as high-risk sites for OSCC development.^43^ These findings highlight the importance of early detection and timely intervention, particularly through targeted screening of these high-risk oral sites.

Saiki et al. (2018) highlighted the crucial role of ALDH3A1 in protecting salivary stem/progenitor cells (SSPCs) from radiation-induced damage, which is essential for salivary gland regeneration. They demonstrated that radiation exposure led to the accumulation of toxic aldehydes, causing DNA, protein, and lipid damage, ultimately resulting in SSPC apoptosis. In particular, the activation of ALDH3A1 through d-limonene significantly reduced aldehyde levels, improved SSPC survival, and preserved salivary gland function in vivo, as well as maintaining the anticancer efficacy of radiation.^44^ These findings agree with our results and support the protective roles of ALDH3A1 in OSCC and its importance as a therapeutic target.

In the present study, we showed that the expression level of ALDH3A1 was low in OSCC tumors with poor differentiation and nodal metastasis, thereby supporting a role for ALDH3A1 in the propagation of tumor dedifferentiation and metastatic behavior. Tumors with advanced pT stages and lymph node metastasis had a higher frequency of weak ALDH3A1 expression. These results suggest that reduced ALDH3A1 expression could enhance tumor progression and metastasis. However, further validation is required in larger and multicenter cohorts as well in systematic studies before ALDH3A1 can be employed as a prognostic biomarker in OSCC.

Our results provide valuable insights into the expression of ALDH3A1 in OSCC and its correlations with tumor pathological stage, histological grade, and lymph node metastasis. However, our study also had the following limitations.

1. The sample size was relatively small and derived from a single-center cohort, which may restrict the generalizability of our findings. A multicenter study with a larger and more diverse population would enhance the reliability of these results.
2. In addition, we focused solely on tumor tissue, and thus assessing ALDH3A1 levels in blood or serum could further support its potential use as a non-invasive biomarker.
3. Moreover, complementing immunohistochemistry with other techniques such as western blotting or quantitative PCR could provide a more comprehensive understanding of the role of ALDH3A1 in OSCC.

## Conclusion

Our results demonstrate that ALDH3A1 has a complex role in OSCC progression. Lower expression levels of ALDH3A1 were linked with advancing tumor grade and stages, and the metastatic spread of lymph nodes. Thus, ALDH3A1 could serve as a marker for predicting the course of OSCC. However, more research is needed to understand how ALDH3A1 functions in OSCC and how it can be used for diagnosis or treatment, thereby potentially helping us to manage OSCC better and improve the outcomes for patients. Nevertheless, the nature of this study is observational and our conclusions are based on immunohistochemistry alone, and should be considered preliminary. Further validation is necessary to confirm the functional role of ALDH3A1 in OSCC pathogenesis by using molecular approaches such as quantitative PCR and western blotting. Future research based on a combination of these techniques may validate ALDH3A1 as a reliable biomarker and therapeutic target in OSCC to assist diagnosis, prognosis, and patient management in the future.

## Ethical approval

In this study, all techniques performed using human samples were undertaken after obtaining approval from the ethical committee of the Institutional Review Board (IRB) of Dow University of Health Sciences (DUHS). **IRB Number: IRB- 2566/DUHS/Approval/2022/1007**.

## Consent

Patients were given thorough explanations of the purpose of the study. Written consent was acquired from patients who were willing to participate.

## Authors contributions

RFK contributed by performing the procedures, acquiring data, and drafting the manuscript. SAA and UB contributed by analyzing and interpreting data. MT also helped with drafting the manuscript. SF helped with sample collection and drafting the manuscript. AAM contributed to statistical analysis of data and interpreting the results. The final text was thoroughly examined and authorized by all authors, who are also responsible for the manuscript's content and similarity index.

## Declaration of Generative AI and AI-assisted technologies in the writing process

In this manuscript, AI and AI-assisted technology were used to improve the readability and language.

## Source of funding

This research did not receive any specific grant from funding agencies in the public, commercial, or not-for-profit sectors.

## Conflict of interest

The authors have no conflicts of interest to declare.

## References

[1] Bray F., Laversanne M., Sung H., Ferlay J., Siegel R.L., Soerjomataram I. (2024). Global cancer statistics 2022: GLOBOCAN estimates of incidence and mortality worldwide for 36 cancers in 185 countries. CA: Cancer J Clin.

[2] Miranda-Filho A., Bray F. (2020). Global patterns and trends in cancers of the lip, tongue and mouth. Oral Oncol.

[3] Filho A.M., Warnakulasuriya S. (2024). Epidemiology of oral cancer in South and South-East Asia: incidence and mortality. Oral Dis.

[4] Qureshi M.A.K.S., Sharafat S., Quraishy M.S. (2020). Common cancers in Karachi, Pakistan: 2010-2019 cancer data from the dow cancer registry. Pak J Med Sci.

[5] Lin H.-J., Wang X.-L., Tian M.-Y., Li X.-L., Tan H.-Z. (2022). Betel quid chewing and oral potential malignant disorders and the impact of smoking and drinking: a meta-analysis. World J Clin Cases.

[6] Anwar N., Pervez S., Chundriger Q., Awan S., Moatter T., Ali T.S. (2020). Oral cancer: clinicopathological features and associated risk factors in a high risk population presenting to a major tertiary care center in Pakistan. PLoS One.

[7] Ferreira A.K.A., de Carvalho S.H.G., Granville-Garcia A.F., de Santana Sarmento D.J., Agripino G.G., de Abreu M.H.N.G. (2020). Survival and prognostic factors in patients with oral squamous cell carcinoma. Med Oral Patol Oral Cirugía Bucal.

[8] Omar E. (2015). Current concepts and future of noninvasive procedures for diagnosing oral squamous cell carcinoma-a systematic review. Head Face Med.

[9] Yang J., Guo K., Zhang A., Zhu Y., Li W., Yu J. (2023). Survival analysis of age-related oral squamous cell carcinoma: a population study based on SEER. Eur J Med Res.

[10] Carreras-Torras C., Gay-Escoda C. (2015). Techniques for early diagnosis of oral squamous cell carcinoma: systematic review. Med Oral Patol Oral Cirugía Bucal.

[11] Das S., Dey M.K., Devireddy R., Gartia M.R. (2023). Biomarkers in cancer detection, diagnosis, and prognosis. Sensors.

[12] Ferrari P., Scatena C., Ghilli M., Bargagna I., Lorenzini G., Nicolini A. (2022). Molecular mechanisms, biomarkers and emerging therapies for chemotherapy resistant TNBC. Int J Mol Sci.

[13] Rahmouni F., Hamdaoui L., Saoudi M., Badraoui R., Rebai T. (2024). Antioxidant and antiproliferative effects of Teucrium polium extract: computational and in vivo study in rats. Toxicol Mech Methods.

[14] Badraoui R., Saeed M., Bouali N., Hamadou W.S., Elkahoui S., Alam M.J. (2022). Expression profiling of selected immune genes and trabecular microarchitecture in breast cancer skeletal metastases model: effect of α–tocopherol acetate supplementation. Calcif Tissue Int.

[15] Jakubczyk K., Dec K., Kałduńska J., Kawczuga D., Kochman J., Janda K. (2020). Reactive oxygen species-sources, functions, oxidative damage. Pol Merkur Lek: Organ Pol Tow Lek.

[16] Checa J., Aran J.M. (2020). Reactive oxygen species: drivers of physiological and pathological processes. J Inflamm Res.

[17] Huang R., Chen H., Liang J., Li Y., Yang J., Luo C. (2021). Dual role of reactive oxygen species and their application in cancer therapy. J Cancer.

[18] Clark D.W., Palle K. (2016). Aldehyde dehydrogenases in cancer stem cells: potential as therapeutic targets. Ann Transl Med.

[19] Zanoni M., Bravaccini S., Fabbri F., Arienti C. (2022). Emerging roles of aldehyde dehydrogenase isoforms in anti-cancer therapy resistance. Front Med.

[20] Xanthis V., Mantso T., Dimtsi A., Pappa A., Fadouloglou V.E. (2023). Human aldehyde dehydrogenases: a superfamily of similar yet different proteins highly related to cancer. Cancers.

[21] Magrassi L., Pinton G., Luzzi S., Comincini S., Scravaglieri A., Gigliotti V. (2024). A new vista of aldehyde dehydrogenase 1A3 (ALDH1A3): new specific inhibitors and activity-based probes targeting ALDH1A3 dependent pathways in glioblastoma, mesothelioma and other cancers. Cancers.

[22] Jang J.-H., Bruse S., Liu Y., Duffy V., Zhang C., Oyamada N. (2014). Aldehyde dehydrogenase 3A1 protects airway epithelial cells from cigarette smoke-induced DNA damage and cytotoxicity. Free Radic Biol Med.

[23] Qu Y., He Y., Yang Y., Li S., An W., Li Z. (2020). ALDH3A1 acts as a prognostic biomarker and inhibits the epithelial mesenchymal transition of oral squamous cell carcinoma through IL-6/STAT3 signaling pathway. J Cancer.

[24] Giebułtowicz J., Wolinowska R., Sztybor A., Pietrzak M., Wroczyński P., Wierzchowski J. (2009). Salivary aldehyde dehydrogenase: activity towards aromatic aldehydes and comparison with recombinant ALDH3A1. Molecules.

[25] Almangush A., Mäkitie A.A., Triantafyllou A., de Bree R., Strojan P., Rinaldo A. (2020). Staging and grading of oral squamous cell carcinoma: an update. Oral Oncol.

[26] Jenniskens J.C., Offermans K., Samarska I., Fazzi G.E., Simons C.C., Smits K.M. (2021). Validity and reproducibility of immunohistochemical scoring by trained non-pathologists on Tissue MicroArrays. Cancer Epidemiol Biomarkers Prev.

[27] Salam H., Ahmed S., Bari M.F., Bukhari U., Haider G., Najeeb S. (2023). Association of Kaiso and partner proteins in oral squamous cell carcinoma. J Taibah Univ Med Sci.

[28] Muzio G., Maggiora M., Paiuzzi E., Oraldi M., Canuto R.A. (2012). Aldehyde dehydrogenases and cell proliferation. Free Radic Biol Med.

[29] Hedberg J.J., Höög J.-O., Nilsson J.A., Xi Z., Elfwing Å., Grafström R.C. (2000). Expression of alcohol dehydrogenase 3 in tissue and cultured cells from human oral mucosa. Am J Pathol.

[30] Counihan J.L., Wiggenhorn A.L., Anderson K.E., Nomura D.K. (2018). Chemoproteomics-enabled covalent ligand screening reveals ALDH3A1 as a lung cancer therapy target. ACS Chem Biol.

[31] Wu D., Mou Y.-P., Chen K., Cai J.-Q., Zhou Y.-C., Pan Y. (2016). Aldehyde dehydrogenase 3A1 is robustly upregulated in gastric cancer stem-like cells and associated with tumorigenesis. Int J Oncol.

[32] Gasparetto M., Smith C.A. (2017). ALDHs in normal and malignant hematopoietic cells: potential new avenues for treatment of AML and other blood cancers. Chem Biol Interact.

[33] Ding Y., Yang M., She S., Min H., Xv X., Ran X. (2015). iTRAQ-based quantitative proteomic analysis of cervical cancer. Int J Oncol.

[34] Ma I., Allan A.L. (2011). The role of human aldehyde dehydrogenase in normal and cancer stem cells. Stem Cell Rev Rep.

[35] Fan F., Yin R., Wang L., Zhao S., Lv D., Yang K. (2021). ALDH3A1 driving tumor metastasis is mediated by p53/BAG1 in lung adenocarcinoma. J Cancer.

[36] Sun H., Zhang M., Li L., Huang Z. (2020). ALDH3B1 is an independent prognostic biomarker of lung adenocarcinoma. Technol Cancer Res Treat.

[37] Luo S., Long H., Lou F., Liu Y., Wang H., Pu J. (2024). Chronic restraint stress promotes oral squamous cell carcinoma development by inhibiting ALDH3A1 via stress response hormone. BMC Oral Health.

[38] He Y., Qu Y., Jin S., Zhang Y., Qin L. (2024). ALDH3A1 upregulation inhibits neutrophils N2 polarization and halts oral cancer growth. Oral Dis.

[39] Ullah A., Pervez N., Javaid M.M., Din S.U., Liaqat S., Junaid M. (2023). Assessment of the influence of risk factors on the incidence of oral squamous cell carcinoma (OSCC) in the northern Pakistani population. Pak J Public Health.

[40] Bhuyan R., Bhuyan S., Panda N., Mohanty J. (2022). Age and gender demographic and statistical analysis in oral squamous cell carcinoma in Eastern India. J Assoc Med Sci.

[41] Mohanta A., Mohanty P.K., Parida G. (2014). Pattern of keratinization in oral squamous cells during carcinogenesis. IOSR J Dent Med Sci.

[42] Sharma J., Deo S., Thulkar S., Iyer V., Pathak M., Bhoriwal S. (2023). Clinicoradiological versus pathological neck staging in locally advanced oral cavity cancers. Oral Oncol Rep.

[43] Gupta A.A., Kheur S., Varadarajan S., Parveen S., Dewan H., Alhazmi Y.A. (2021). Chronic mechanical irritation and oral squamous cell carcinoma: a systematic review and meta-analysis. Bosn J Basic Med Sci.

[44] Saiki J.P., Cao H., Van Wassenhove L.D., Viswanathan V., Bloomstein J., Nambiar D.K. (2018). Aldehyde dehydrogenase 3A1 activation prevents radiation-induced xerostomia by protecting salivary stem cells from toxic aldehydes. Proc Natl Acad Sci.

